# 

**Published:** 1910-12-15

**Authors:** 


### ANNOUNCEMENTS.

INSTITUTE OF DENTAL PEDAGOGICS.—The annual meeting of this society will be held in Washington City, D. C., December 27th to 29th, 1910. A programme full of interest to all teachers and practitioners is being prepared and all interested dentists will be welcomed.

JOHN Q. BYRAM, *President.*

Marquette University —The Alumni Association of the Dental Department of this University will be hold its fifth annual reunion in the Auditorium at Milwaukee, January 24th and 25th, 1911. All alumni are cordially invited to attend.

O. G. Krause, D.D.S., *Secretary*.

---

The G. V. Black Dental Club of St. Paul. This Society will hold its meeting in February and the officers are making a great effort to make it the best meeting the club has ever held. For programmes and date address Dr. R. B. Wilson, American National Bank Building, St. Paul, Minn.

---

Michigan State Dental Society. The next meeting of this society will be held at Grand Rapids during the week beginning the 9th. The change of time of this meeting from July to April is to meet the wishes of many dentists who do not like to have their summer vacations interfered with by this meeting, and to others who desire to attend other important meetings which are held at times so close to the former meeting time as to make it difficult for some to attend both meetings. It is hoped there may be a large attendance out so that the wisdom of this move may be verified, or if not satisfactory that another time more favorable might be selected.

